# Association of CXCL10 and CXCL13 levels with disease activity and cutaneous manifestation in active adult-onset Still’s disease

**DOI:** 10.1186/s13075-015-0773-4

**Published:** 2015-09-19

**Authors:** Jae Ho Han, Chang-Hee Suh, Ju-Yang Jung, Jin-Young Nam, Jee Eun Kwon, Hyunee Yim, Hyoun-Ah Kim

**Affiliations:** Department of Pathology, Ajou University School of Medicine, 164 Worldcup-ro, Yeongtong-gu, Suwon, 443-380 South Korea; Department of Rheumatology, Ajou University School of Medicine, 164 Worldcup-ro, Yeongtong-gu, Suwon, 443-380 South Korea

## Abstract

**Introduction:**

C-X-C motif chemokine 10 (CXCL10) is produced in response to interferon-γ, and tumor necrosis factor-α (TNF-α) triggers the accumulation of activated lymphocytes. CXCL13 is constitutively expressed in secondary lymphoid tissues, and the expression is upregulated by TNF-α, via T cell stimulation. It appears that CXCL10 and CXCL13 could play a potential role in the pathogenesis of adult-onset Still’s disease (AOSD), therefore, we investigated the associations between CXCL10 and CXCL13 levels and clinical manifestations in patients with active AOSD.

**Methods:**

Blood samples were collected from 39 active AOSD patients, 32 rheumatoid arthritis (RA) patients and 40 healthy controls (HC). Of the AOSD patients, follow-up samples were collected from 15 9.6 ± 9.2 months later. Serum levels of CXCL10 and CXCL13 were determined using enzyme-linked immunosorbent assay. CXCL10, CXCL13, and C-X-C chemokine receptor type 3 (CXCR3) expression levels in biopsy specimens obtained from 26 AOSD patients with skin rashes were investigated via immunohistochemistry.

**Results:**

The CXCL10 levels in AOSD patients (1,031.3 ± 2,019.6 pg/mL) were higher than in RA (146.3 ± 91.4 pg/mL, *p* = 0.008) and HC (104.4 ± 47.9 pg/mL, *p* = 0.006). Also, the CXCL13 levels of AOSD patients (158.8 ± 151.2 pg/mL) were higher than those of RA (54.4 ± 61.1 pg/mL, *p* < 0.001) and HC (23.5 ± 18.1 pg/mL, *p* < 0.001). Serum CXCL10 levels correlated with ferritin and systemic scores. Serum CXCL13 levels correlated with those of hemoglobin, C-reactive protein, ferritin, and albumin, and systemic scores. In follow-up AOSD patients, the levels of CXCL10 and CXCL13 fell significantly (153.7 ± 130.1 pg/mL, *p* = 0.002, and 89.1 ± 117.4 pg/mL, *p* = 0.001, respectively). On immunohistochemistry, the percentages of inflammatory cells expressing CXCL10 ranged from 1 to 85 %, CXCL13 from 1 to 72 %, and CXCR3 from 2 to 65 %. The percentage of CXCL10-positive inflammatory cells was higher in skin biopsy samples exhibiting mucin deposition than in those that did not (*p* = 0.01). CXCL13 levels were correlated with those of CD4 and CD68.

**Conclusions:**

Serum CXCL10 and CXCL13 levels may serve as clinical markers for assessment of disease activity in AOSD. CXCL10/CXCR3 and CXCL13 may contribute to the inflammatory response, especially skin manifestations thereof, in AOSD.

**Electronic supplementary material:**

The online version of this article (doi:10.1186/s13075-015-0773-4) contains supplementary material, which is available to authorized users.

## Introduction

Adult-onset Still’s disease (AOSD) is a systemic inflammatory disease exhibiting various clinical manifestations characterized by a high spiking fever, an evanescent skin rash, polyarthralgia, and hepatosplenomegaly [1, 2]. Acute-phase reactants, such as the C-reactive protein (CRP) and ferritin levels, erythrocyte sedimentation rate (ESR), and leukocyte and platelet counts, are typically elevated. Although initial manifestations (the systemic symptoms) are similar in all patients, the clinical courses differ; several patterns are evident. These include systemic monocyclic, intermittent or polycyclic, and chronic articular, patterns [2–4]. Although the pathogenesis of AOSD remains unknown, several factors including infections, genetic factors, and immune dysregulation have been suggested to contribute to development of the disease [5, 6]. Several studies with small case numbers have reported associations between distinct human leukocyte antigens alleles and AOSD, but the data are often conflicting, varying with ethnicity and/or disease pattern [7]. Several cytokines including interleukin-1 (IL-1), tumor necrosis factor-α (TNF-α), IL-18, IL-8, and IL-6 have been suggested to play roles in the pathogenesis of AOSD [8, 9].

As is true of other cytokines, the levels of several chemokines have been reported to change under various systemic conditions, and such changes may serve as useful diagnostic and prognostic markers [10]. It appears that proinflammatory chemokines may play roles in AOSD pathogenesis characterized by various systemic symptoms, but studies on such chemokines are limited in number. Chemokines are relatively small-sized (8–14 kDa) chemotactic cytokines produced by a variety of cells, and are characterized by the presence of three to four conserved cysteine residues [10, 11]. Proinflammatory chemokines are produced by cells primarily to recruit leukocytes to the sites of inflammation or infection. IL-8, or C-X-C motif chemokine 8 (CXCL8), mobilizes, activates, and degranulates neutrophils at sites of inflammation. The IL-8 levels have been found to be elevated in AOSD patients, and may serve as a biomarker predicting the persistence of arthritis [8, 9]. A recent study showed that the levels of C-X3-C motif ligand 1, CXCL8, CXCL10, CC-chemokine ligand 2 (CCL2), and CCL3 in the sera of 19 AOSD patients were higher than those of healthy controls (HC) [12].

CXCL10 is produced in response to interferon-γ (IFN-γ), and TNF-α triggers the accumulation of activated lymphocytes via interaction with a specific receptor, C-X-C chemokine receptor type 3 (CXCR3), creating an amplification feedback loop [13, 14]. CXCL10 is secreted by several cell types, including T cells, monocytes, neutrophils, and endothelial cells. CXCL13 is constitutively expressed by follicular dendritic cells in secondary lymphoid tissues, and is required for lymphoid follicle formation. The expression thereof is upregulated by TNF-α, via T cell stimulation, and CXCR3 and CXCR5 are known to serve as receptors for CXCL13 [11, 15]. CXCL13 is also associated with formation of extranodal lymphoid aggregates, and attracts B cells to secondary lymphoid tissue, facilitating local inflammation and antibody generation [15, 16]. Therefore, CXCL13 is thought to be a pivotal chemokine in establishment of the adaptive immune response. These chemokines and their receptors seem to be involved in several inflammatory diseases, including rheumatoid arthritis (RA), systemic lupus erythematosus (SLE), and inflammatory bowel disease [14, 17–24]. Furthermore, a recent study showed that injury-induced S100A8/A9 stimulated CXCL10 production in monocytes and macrophages [25]. Previously, we showed that S100A8/A9 levels were elevated in AOSD patients compared to HC, and S100A8/A9 levels correlated with those of AOSD disease activity markers [26]. Therefore, the aim of the present study was to determine the serum levels of CXCL10 and CXCL13, and to investigate their associations with clinical activities, in patients with active AOSD. To explore the in vivo involvement of these chemokines in AOSD, we performed immunohistochemical analysis of skin biopsy for CXCL10, CXCL13, and their receptor, CXCR3 in 26 patients with active untreated AOSD.

## Materials and methods

### Subjects

Thirty-nine active AOSD patients, 32 RA patients and 40 HC were included in the present study, and serum samples were collected from all subjects. AOSD patients were diagnosed using Yamaguchi’s criteria after exclusion of those who had infectious, neoplastic and autoimmune disorders [27]. Twenty-one patients of AOSD were evaluated with tuberculosis skin test or *Mycobacterium tuberculosis*-specific T cell IFN-γ assays before starting high-dose glucocorticosteroid therapy, and only two patients had latent tuberculosis. The HC was recruited using a screening questionnaire from healthy individuals without history of exposure to *Mycobacterium tuberculosis* and medical history of autoimmune, rheumatic, and any other diseases through the public announcement. Of the 39 AOSD patients, follow-up samples were collected from 15 9.6 ± 9.2 months later. All blood samples were stored at −70 °C immediately after collection. Information on medical histories, clinical symptoms, and the findings of physical examinations was entered into a database together with serum test results. Each patient underwent a series of laboratory tests, including a complete blood count; ESR evaluation; assessment of CRP, rheumatoid factor (RF), anti-nuclear antibody (ANA), and ferritin levels (normal 13–150 ng/mL for females and 30–400 ng/mL for males); liver function testing; and urinalysis. AOSD disease activity was scored as previously described; scores ranged from 0 to 12 with 1 point being given for each of the following manifestations: fever, a typical rash, pleuritis, pneumonia, pericarditis, hepatomegaly or abnormal liver function test data, splenomegaly, lymphadenopathy, leukocytosis ≥ 15,000/mm^2^, sore throat, myalgia, and abdominal pain [28]. This study was approved by Ajou University Hospital Institutional Review Board (IRB No. BMR-KSP-14-406), and informed consent was obtained from all subjects.

### CXCL10 and CXCL13 assays

CXCL10 levels were measured using commercial enzyme-linked immunosorbent assay (ELISA) kits (R & D Systems, Minneapolis, MN, USA) according to the manufacturer’s instructions. CXCL13 levels were also determined using an ELISA kit (R & D Systems) according to the manufacturer’s instructions.

### Histopathological analysis of skin biopsy sample

Skin biopsies were obtained from 26 patients with AOSD, and we examined hematoxylin and eosin-stained sections. All slides were independently examined by three pathologists (JHH, JEK, and HY) with respect to the following skin histological parameters: (1) epidermal change; (2) extent of inflammatory cell infiltration; and the presence of (3) karyorrhexis, (4) vasculitis, and (5) interstitial mucin.

### Immunohistochemical evaluation

Immunohistochemistry were performed on the formalin-fixed paraffin-embedded sections using a Benchmark XT automated staining system (Ventana Medical Systems Inc., Tucson, AZ, USA). The primary antibodies used were: CD4, 1:10 and CD8, 1:50 (Thermo Fisher Scientific, Fremont, CA, USA); CD68, 1:200 (Novocastra Laboratories Ltd, Newcastle, UK); CXCL10, 1:50; CXCL13, 1:50; and CXCR3, 1:20 (R & D Systems). Detection was achieved using a Ventana Optiview DAB kit (Ventana Medical Systems). Scores were calculated by dividing the numbers of positive inflammatory cells by the numbers of all inflammatory cells, expressed as percentages (CXCL10, CXCL13, and CXCR3) or graded on a scale from 1 to 3: 1:1–33 %; 2, 34–66 %; and 3, 67–100 % (CD4, CD8, and CD68).

### Statistical analyses

All data are shown as means ± standard deviations. Differences among CXCL10 and CXCL13 levels were evaluated using the independent *t* test or the Mann–Whitney *U* test. Correlations between histological scores and disease activity marker levels were evaluated using Pearson’s correlation test or Spearman’s correlation test. The Wilcoxon signed-rank test was also used to compare CXCL10 and CXCL13 levels in patients who underwent follow-up serum sampling. All statistical analyses were performed using SPSS version 20.0 (IBM Corp., Armonk, NY, USA). A *p* value <0.05 was regarded as indicative of statistical significance.

## Results

### Clinical characteristics of the patients

Table 1 summarizes the clinical characteristics of the 39 patients with AOSD, RA and the HC. The mean age of AOSD patients was 42.2 ± 15.8 years and females comprised 92.3 % of all patients. There was no significant difference in age or gender between the groups except age between RA and HC (*p* = 0.001). The principal clinical symptoms of AOSD patients included a high spiking fever (87.2 %), a skin rash (82.1 %), arthritis (61.5 %), sore throat (53.8 %), and splenomegaly (25.6 %). In RF-positive patients, a diagnosis of RA was excluded using the 1987 American College of Rheumatology (ACR) criteria [29]. In ANA-positive patients, a diagnosis of SLE was excluded using the 1982 ACR criteria [30]. Of the 39 AOSD patients, 30 were in the initial stages of high-level disease activity before commencement of treatment. The other nine were in flare during follow-up of AOSD, and their disease duration was 28.2 ± 23.2 months. Among the nine patients, three patients were discontinued with their medications before the flare. Five patients were treated with methotrexate, and one patient was treated with azathioprine at the time of sampling. Daily glucocorticosteroid dose of the six AOSD patients was 5.3 ± 6.3 mg prednisolone equivalent. Nineteen patients had AOSD of the monophasic pattern, six disease of the polycyclic pattern, and five disease of the chronic articular pattern, among 30 patients exhibiting high-level disease activity prior to commencement of treatment (thus at the time of sampling). Disease duration of the RA patients was 41.2 ± 26.3 months. In RA patients, 21 patients (65.6 %) were treated with methotrexate at the time of sampling, four (12.5 %) with sulfasalazine, six (18.8 %) with leflunomide, and one patient with adalimumab (3.1 %), respectively. Daily glucocorticosteroid dose of the RA patients was 2.23 ± 2.

**Table 1:** Clinical characteristics of patients

	AOSD (*n* = 39)	RA (*n* = 32)	HC (*n* = 40)
Age (years)	42.2 ± 15.8	44.7 ± 8	37.7± 8.7
Gender (F/M)	36/3	27/5	35/5
Fever	34 (87.2)		
Sore throat	21 (53.8)		
Skin rash	32 (82.1)		
Lymphadenopathy	14 (35.9)		
Splenomegaly	10 (25.6)		
Hepatomegaly	5 (12.8)		
Pericarditis	6 (15.4)		
Arthritis	24 (61.5)	31 (96.9)	
Hemoglobin, g/dL	11 ± 1.8	12.8 ± 1.4	
Leukocytes, /μL	13,130 ± 5,015	7,613 ± 3,061	
Platelets, x10^3^/μL	316.2 ± 117.9	267.9 ± 78.9	
Ferritin, ng/mL	7,229.7 ± 13,091.1		
ESR, mm/h	64.6 ± 29.3	31.9 ± 24.7	
CRP, mg/dL	8.53 ± 6.68	1.07 ± 2.41	
AST/ALT, mg/dL	55.8 ± 42.8/54.4 ± 46.7	25.2 ± 10.3/23.3 ± 17.4	
Bilirubin, mg/dL	0.67 ± 0.8	0.58 ± 0.2	
Albumin, g/dL	3.74 ± 0.64	4.48 ± 0.32	
ANA positivity	6 (15.4)	9 (28.1)	
RF positivity	4 (10.3)	29 (90.6)	
Systemic score	4.79 ± 1.81		
DAS-28		3.91 ± 1.2	

*AOSD* adult onset Still’s disease, *RA* rheumatoid arthritis, *HC* healthy controls, *ESR* erythrocyte sedimentation rate, *CRP* C-reactive protein, *AST* aspartate transaminase, *ALT* alanine transaminase, *ANA* antinuclear antibody, *RF* rheumatoid factor. DAS-28, disease activity score including 28 joints. All values are presented as numbers (with percentages) or means ± standard deviations. The systemic scoring system of Pouchot et al. [28] assigns a score from 0 to 12 with 1 point for each of the following manifestations: fever, typical rash, pleuritis, pneumonia, pericarditis, hepatomegaly or abnormal liver function test data, splenomegaly, lymphadenopathy, leukocytosis ≥ 15,000/mm^2^, sore throat, myalgia, and abdominal pain

Of the AOSD patients, five (12.8 %) were diagnosed with reactive hemophagocytic syndrome (RHS). These patients were diagnosed to RHS by tissue biopsy or clinical features [31].

### Serum CXCL10 and CXCL13 levels

**Fig. 1:**
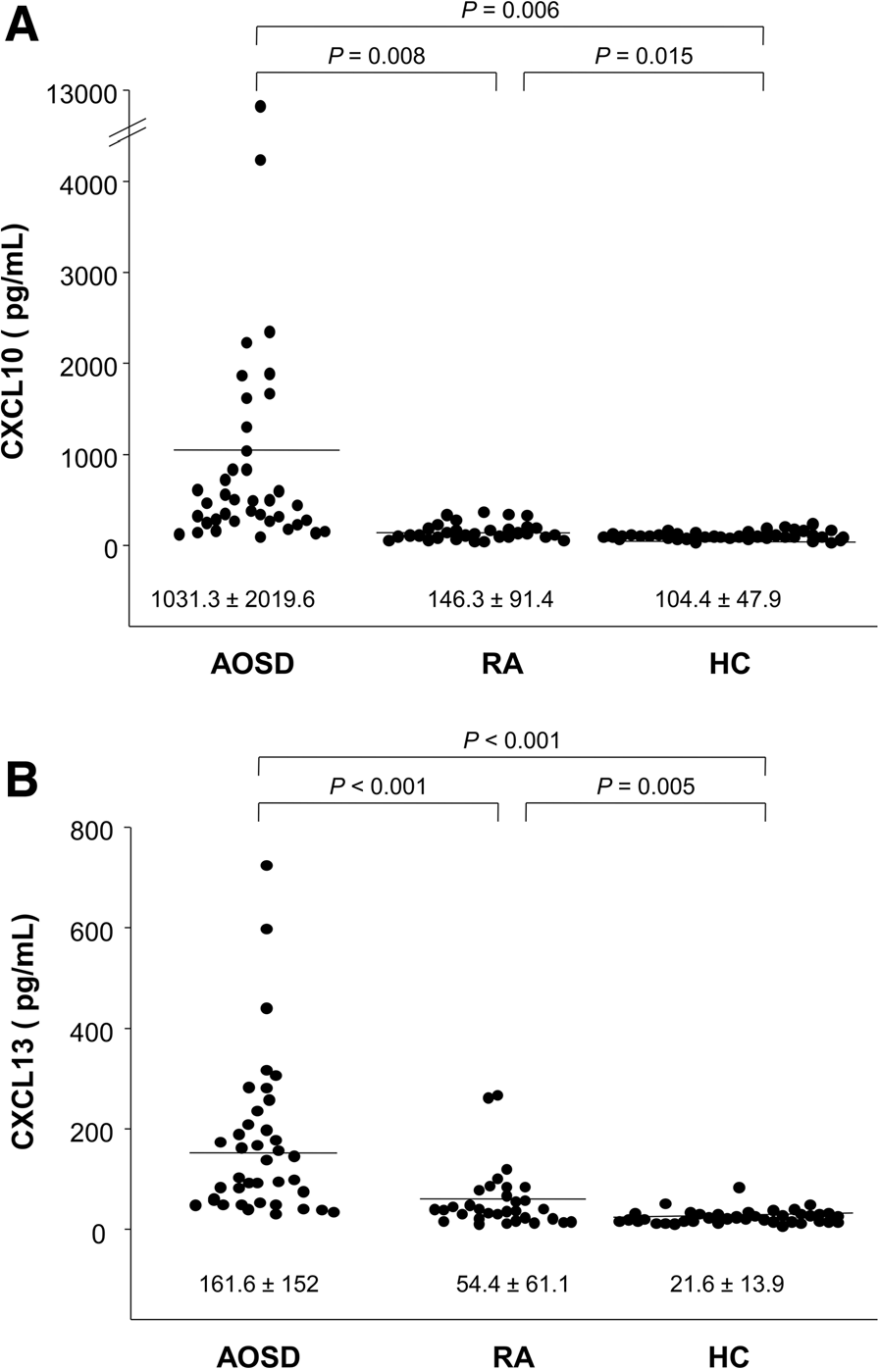
The levels of CXCL10 (**a**) and CXCL13 (**b**) in 39 adult-onset Still’s disease (AOSD) patients, 32 rheumatoid arthritis (RA) patients and 40 healthy controls (HC). Data are expressed as means ± SDs. The independent *t* test was used to perform statistical analysis. *CXCL10*, C-X-C motif chemokine 10, *CXCL13*, C-X-C motif chemokine 13

Figure 1 shows the CXCL10 and CXCL13 levels in AOSD patients, RA patients and HC. The CXCL10 levels in AOSD patients (1,031.3 ± 2,019.6 pg/mL) were higher than in RA (146.3 ± 91.4 pg/mL, *p* = 0.008) and HC (104.4 ± 47.9 pg/mL, *p* = 0.006) (Fig. 1a). Also, the CXCL13 levels of AOSD patients (158.8 ± 151.2 pg/mL) were higher than those of RA (54.4 ± 61.1 pg/mL, *p* < 0.001) and HC (23.5 ± 18.1 pg/mL, *p* < 0.001) (Fig. 1b). We compared the CXCL10 and CXCL13 levels between patients with initially active AOSD and those experiencing flares during follow-up. The CXCL10 levels of the 30 initially active AOSD patients (1,309.5 ± 2,272.1 pg/mL) were higher than those of the nine flare patients (204.3 ± 90 pg/mL, *p* < 0.001). However, the CXCL13 levels did not differ between the two groups (163 ± 147.6 pg/mL vs. 157 ± 175.4 pg/mL, *p* = 0.635).

Comparing the CXCL10 and CXCL13 levels according to existence of RHS, we found that serum CXCL13 were significantly higher in AOSD patients with RHS (229.6 ± 90.3 pg/mL) than in patients without RHS (151.6 ± 157.6 pg/mL, *p* = 0.039), but serum CXCL10 did not differ between the two groups. When we compared CXCL10 and CXCL13 levels by AOSD clinical course (monocyclic vs. polycyclic vs. chronic articular) of 30 patients with high-level disease activity, those levels did not differ among the three groups (data not shown).

### Correlation between serum CXCL10 or CXCL13 levels and disease activity in AOSD patients

The correlations between the levels of pre-existent disease activity markers and those of serum CXCL10 or CXCL13 in AOSD patients are shown in Table 2. Serum CXCL10 levels correlated with ferritin levels (*r* = 0.444, *p* = 0.005), aspartate transaminase (AST) levels (*r* = 0.412, *p* = 0.009), and systemic scores (*r* = 0.421, *p* = 0.008). Serum CXCL13 levels correlated with hemoglobin levels (*r* = −0.464, *p* = 0.003), CRP levels (*r* = 0.534, *p* < 0.001), ferritin levels (*r* = 0.405, *p* = 0.011), albumin levels (*r* = −0.398, *p* = 0.012), and the systemic scores (*r* = 0.809, *p* < 0.001). Also, serum CXCL10 levels correlated with those of CXCL13 (*r* = 0.428, *p* = 0.007).

**Table 2:** Correlations between C-X-C motif chemokine 10 (CXCL10) and CXCL13 levels and those of disease activity markers in 39 adult-onset Still’s disease patients

Disease activity marker	Correlation coefficient, r (*p*-value)		
	CXCL10	CXCL13	Systemic score
Systemic score	0.421 (0.008)	0.809 (<0.001)	
Leukocytes	0.052 (0.753)	0.21 (0.2)	0.362 (0.024)
Hemoglobin	−0.137 (0.406)	−0.464 (0.003)	−0.3 (0.063)
Platelets	−0.021 (0.899)	0.09 (0.587)	0.042 (0.8)
ESR	−0.132 (0.423)	0.11 (0.504)	0.191 (0.244)
CRP	0.147 (0.373)	0.534 (<0.001)	0.619 (<0.001)
Ferritin	0.444 (0.005)	0.405 (0.011)	0.5 (0.001)
Albumin	−0.216 (0.187)	−0.398 (0.012)	−0.475 (0.002)
Bilirubin	−0.116 (0.482)	−0.369 (0.021)	−0.392 (0.013)
AST	0.412 (0.009)	0.162 (0.325)	0.301 (0.062)
ALT	0.3 (0.064)	0.06 (0.715)	0.209 (0.202)
CXCL10		0.428 (0.007)	0.421 (0.008)
CXCL13	0.428 (0.007)		0.809 (<0.001)

*ESR* erythrocyte sedimentation rate, *CRP* C-reactive protein, *AST* aspartate transaminase, *ALT* alanine transaminase. Spearman’s correlation coefficients were calculated. The systemic scoring system of Pouchot et al. [28] assigns a score from 0 to 12 with 1 point for each of the following manifestations: fever, typical rash, pleuritis, pneumonia, pericarditis, hepatomegaly or abnormal liver function test data, splenomegaly, lymphadenopathy, leukocytosis ≥ 15,000/mm^2^, sore throat, myalgia, and abdominal pain

Furthermore, we compared the correlation coefficient between systemic scores and several inflammatory markers including CXCL10 and CXCL13. Systemic scores have the best correlation coefficient with CXCL13 levels (*r* = 0.809) than CRP (*r* = 0.619, *p* < 0.001), ferritin (*r* = 0.5, *p* = 0.001), CXCL10 (*r* = 0.0421, *p* = 0.008), and ESR (*r* = 0.191, *p* = 0.244).

### Changes in serum CXCL10 and CXCL13 levels in follow-up AOSD patients

**Fig. 2:**
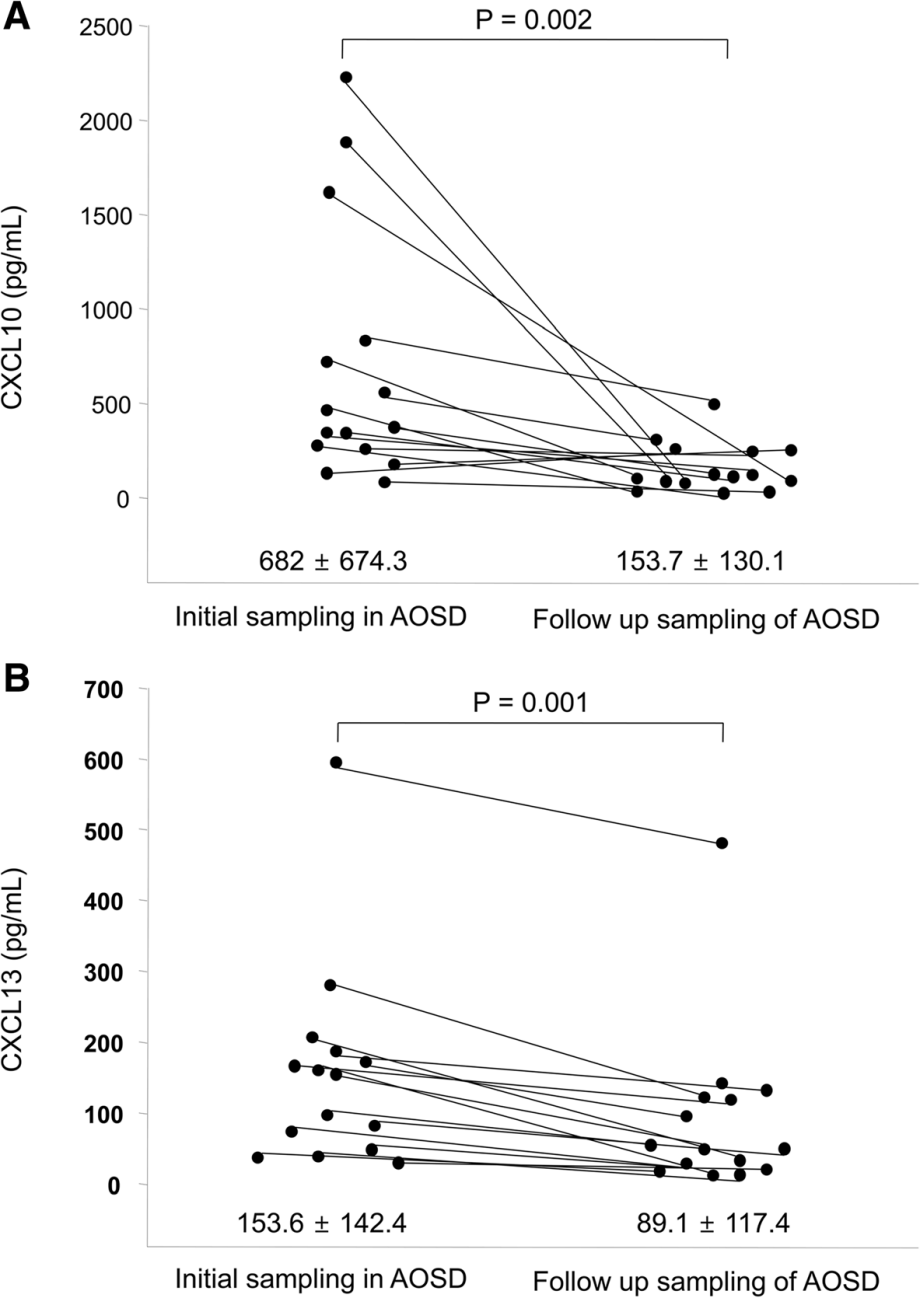
The levels of CXCL10 (**a**) and CXCL13 (**b**) in 15 follow-up adult-onset Still’s disease (AOSD) patients. Data are expressed as means ± SDs. The Wilcoxon signed-rank test was used to perform statistical analysis. *CXCL10*, C-X-C motif chemokine 10, *CXCL13* C-X-C motif chemokine 13

Repeat serum samples were collected from 15 AOSD patients at 9.6 ± 9.2 months after the first samplings. The CXCL10 level was 153.7 ± 130.1 pg/mL, and that of CXCL13 89.1 ± 117.4 pg/mL. The systemic scores were 1.13 ± 1.19. In AOSD patients who were followed up, systemic scores decreased somewhat and serum CXCL10 and CXCL13 levels significantly decreased (*p* = 0.002 and *p* = 0.001) (Fig. 2). We calculated correlations between changes in CXCL10 or CXCL13 levels and changes in disease activity marker levels (Table 3). The change in CXCL10 was positively correlated only with the change in systemic score (*r* = 0.516, *p* = 0.049). The change in CXCL13 was positively correlated with both the change in CRP level (*r* = 0.604, *p* = 0.017) and the change in systemic score (*r* = 0.642, *p* = 0.01).

**Table 3:** Correlations between changes in serum C-X-C motif chemokine 10 (CXCL10) or CXCL13 levels and those of disease activity markers in follow-up adult-onset Still’s disease patients

Disease activity marker	Correlation coefficient, r (*p*-value)	
	Delta CXCL10	Delta CXCL13
Delta systemic score	0.516 (0.049)	0.642 (0.01)
Delta leukocytes	0.036 (0.899)	0.356(0.193)
Delta hemoglobin	−0.175 (0.532)	0.177 (0.528)
Delta platelets	0.289 (0.296)	0.036 (0.899)
Delta ESR	0.029 (0.919)	0.175 (0.533)
Delta CRP	0.05 (0.86)	0.604 (0.017)
Delta ferritin	0.35 (0.201)	0.425 (0.114)
Delta albumin	−0.136 (0.628)	0.142 (0.614)
Delta bilirubin	0.061 (0.829)	−0.106 (0.707)
Delta AST	0.438 (0.103)	−0.116 (0.68)
Delta ALT	0.464 (0.081)	−0.468 (0.079)

*ESR* erythrocyte sedimentation rate, *CRP* C-reactive protein, *AST* aspartate transaminase, *ALT* alanine transaminase. Spearman’s correlation coefficients were calculated. The systemic scoring system of Pouchot et al. [28] assigns a score from 0 to 12 with 1 point for each of the following manifestations: fever, typical rash, pleuritis, pneumonia, pericarditis, hepatomegaly or abnormal liver function test data, splenomegaly, lymphadenopathy, leukocytosis ≥ 15,000/mm^2^, sore throat, myalgia, and abdominal pain

### Histopathological characteristics of skin

The skin manifestations of 17 patients were maculopapular eruptions on the upper and lower extremities, and the trunk (*n* = 17, 65.4 %). Five patients (19.2 %) had persistent pruritic eruptions, and two patients had papulopustular lesions on the trunk. Two patients had painful swelling of the low extremities. Most skin biopsies exhibited mild lymphocytic or histiocytic infiltration in the upper dermis. Nuclear debris was frequently evident in the dermis (14 cases; 53.8 %). Neutrophil infiltrations were shown in some cases (seven cases; 26.9 %), More than half of all cases showed interstitial mucin deposition. Some cases exhibited interface dermatitis with keratinocyte necrosis or basal vacuolization.

### Immunohistochemical data

**Fig. 3:**
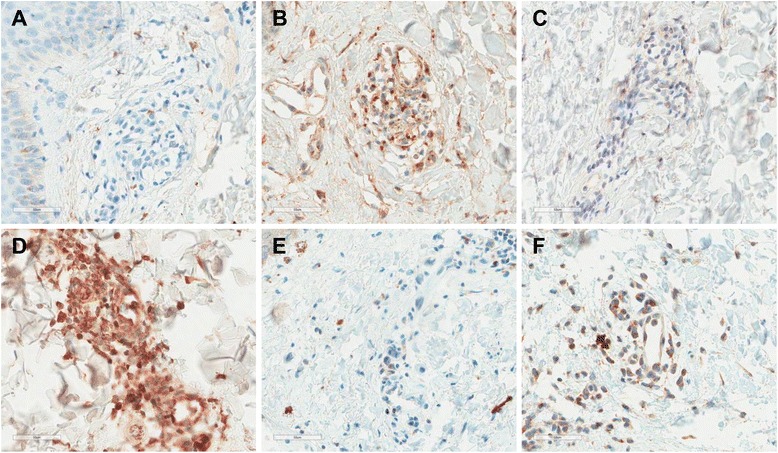
CXCL10 (**a**, **b**), CXCL13 (**c**, **d**), and CXCR3 (**e**, **f**) expression in inflammatory cells in skin biopsy material of patients with adult-onset Still’s disease (original magnification, ×400). Representative instances of rare expression (**a**, **c**, **e**) and frequent expression (**b**, **d**, **f**) are shown. *CXCL10*, C-X-C motif chemokine 10, *CXCL13* C-X-C motif chemokine 13, *CXCR3* C-X-C chemokine receptor type 3

The percentage of cells positive for CD8 was higher than that for CD68 (57.7 %). The number of CD8-positive lymphocytes was higher than that of CD4-positive lymphocytes in almost all cases (88.5 %). To control for CXCL10, CXCL13, and CXCR3 immunohistochemical evaluations, lymphoid cells in the paracortical zone or germinal center of a reactive lymph node were stained. The antibodies revealed a granular pattern of cytoplasmic staining. The staining patterns of inflammatory cells in skin biopsies were similar to those of lymphoid cells in a lymph node (Fig. 3). The percentages of inflammatory cells expressing CXCL10 ranged from 1 to 85 % (mean, 25.8 ± 23.3 %), CXCL13 from 1 to 72 % (mean, 23.3 ± 22.2 %), and CXCR3 from 2 to 65 % (mean, 16.4 ± 18.5 %). The percentages of inflammatory cells expressing CXCL10 and CXCL13 were not correlated with systemic score, ESR, CRP and ferritin. CXCL10-, CXCL13-, and CXCR3-positive inflammatory cells did not differ in terms of the presence of neutrophil infiltrations, keratinocyte vacuolization or karyorrhexis (Additional file 1). However, the percentage of CXCL10-positive inflammatory cells was higher in patients exhibiting mucin deposition than in those who did not (*p* = 0.01).

The correlations between CD4, CD8, and CD68 grades, and the percentages of CXCL10-, CXCR3-, and CXCL13-positive inflammatory cells in the skin of AOSD patients, are shown in Additional file 2. CXCL10 and CXCR3 levels did not correlate with any grade. However, CXCL13 levels were correlated with those of CD4 (*r* = 0.453, *p* = 0.02) and CD68 (*r* = 0.464, *p* = 0.017).

## Discussion

To evaluate the clinical utilities of serum CXCL10 and CXCL13 levels, we studied these markers of AOSD patients and compared the levels with those of RA and HC. Moreover, we also confirmed that these markers and their receptor, CXCR3, immunohistochemically stained in skin rash material from AOSD patients. This is the first study to show that serum CXCL13 levels are significantly higher in patients with active AOSD than in RA and HC. Furthermore, the serum levels of CXCL10 and CXCL13 in patients with AOSD correlated with those of several inflammatory markers, and systemic scores, and the serum levels of CXCL10 and CXCL13 fell upon reduction in disease activity in follow-up AOSD patients.

CXCL10 has been detected in the serum, synovial fluid, and synovial tissues of RA patients, and CXCR3, the receptor thereof, is expressed in T cell-rich areas of inflamed synovial tissue [20, 21, 24, 32, 33]. The elevated levels of CXCR3 in synovial fluid T cells have been associated with high levels of IFN-γ, suggesting that a Th1 phenotype is preferentially in play [32]. Only one study has reported elevated serum CXCL10 levels in 19 patients with AOSD compared to HC [12]. However, CXCL10 levels were not shown to be correlated with those of ferritin, CRP, or IL-18. In our present study, the serum CXCL10 levels of 39 AOSD patients were significantly higher than those of RA and HC, and serum CXCL10 levels were significantly correlated with the levels of known disease activity markers, including ferritin and AST, and the systemic score. Most follow-up AOSD patients exhibited significantly reduced CXCL10 levels after improvement in disease, although changes in CXCL10 levels were correlated only with changes in systemic scores. Furthermore, CXCL10 and the receptor thereof, CXCR3, were expressed in the affected skin of AOSD patients, and enhanced CXCL10 staining was evident in inflammatory cells of skin lesions with mucin depositions. Actually, in lupus erythematosus tumidus, a lupus erythematosus subset characterized by dense perivascular lymphocytic infiltration and mucin deposits, CXCL10 were mainly expressed in the perivascular folds, again reflecting the typical inflammatory pattern [34]. Therefore, such results suggest that CXCL10 plays a role in the skin dermal injury of AOSD, and serum levels may be a good marker for monitoring of disease activity. Large amounts of type 1 inflammatory cytokines, including IFN-γ, TNF-α, and IL-18, are present in the serum and diseased tissues (including the liver and skin) of AOSD patients [9, 26, 35–37]. These cytokines may sustain systemic and local proinflammatory responses and upregulate CXCL10 expression. In turn, CXCL10 may participate in maintenance of the default Th1 polarization noted during AOSD inflammation, because CXCL10 is known to upregulate cytokine synthesis in human Th1 cells [33, 37].

Proinflammatory cytokines, such as TNF-α and IL-6, play roles upstream of CXCL13 expression and lymphoid tissue organization, as demonstrated in animal models of lymphoid tissue generation [38, 39]. Many researchers have measured CXCL13 levels in the sera and synovial tissues of RA patients or mouse models of RA [15, 16, 21–23, 40]. One recent study showed that synovial CXCL13 could serve as a marker of more severe RA [23]. Greison et al. [22] proposed that high CXCL13 concentrations indicated recent-onset inflammation that might respond better to early aggressive treatment. Although acute AOSD patients have similar symptoms, including fever, rash, and myalgia, only some exhibit the chronic articular pattern associated with tissue destruction [3, 36]. Therefore, we hypothesized that CXCL13 might play a key role in triggering the appearance of the chronic articular pattern after systemic disease manifestations, and measured CXCL13 levels in the serum and skin of active AOSD patients. Serum CXCL13 levels in AOSD patients were significantly higher than those of RA and HC, and serum CXCL13 levels strongly correlated with the levels of several disease activity markers, including those of hemoglobin, albumin, AST, CRP, and ferritin; and the systemic score. Furthermore, when we compared the correlation coefficient between systemic scores and several inflammatory markers including CXCL10 and CXCL13, systemic score have the best correlation with CXCL13 levels than CRP, ferritin, CXCL10, and ESR. Most follow-up AOSD patients exhibited significantly decreased CXCL13 levels after improvement in disease activity, and changes in serum CXCL13 levels were always correlated with changes in CRP levels and systemic scores, although the number of follow-up patients was small. Furthermore, we found that serum CXCL13 levels were significantly higher in AOSD patients with RHS than in patients without RHS, and that systemic scores have the best correlation coefficient with CXCL13 levels. These results strongly suggest that CXCL13 plays a significant role in the pathogenesis, clinical manifestation and severity such as RHS in AOSD, and can serve as a useful marker for monitoring disease activity. CXCL13 was also expressed in skin affected by AOSD; variable percentages of inflammatory cells were positive for this material. Such staining was correlated with the numbers of CD4- and CD68-stained inflammatory cells. Although the percentage of the inflammatory cells were CD8+ T cells were higher than that of macrophages and CD4+ T cells, we can suggest that CXCL13 is expressed or secreted by macrophages or CD4+ T cells in the patients with AOSD. CXCL13-producing CD4+ T cells may be a CD4 subset other than Th1, Th2, TH17 or CXCR5+ follicular helper T cells as in chronic inflammatory disorders such as RA [14]. Gene expression profiling has identified genes uniquely expressed by human germinal-center T helper cells [41]. However, we did not find any difference in CXCL13 levels among patients with different AOSD disease patterns. Although a few reports on differences in patient genetic backgrounds, cytokine levels, or clinical features of chronic articular AOSD compared with non-chronic articular or monophasic AOSD, have appeared, studies on the pathogenesis of the various AOSD patterns are few in number [8, 36, 42]. In the present work, the CXCL13 levels did not differ among patients exhibiting different disease courses, but the number of AOSD patients with the chronic articular pattern of disease was small. Also, elevated CXCL13 levels could differentially affect the synovial recruitment of inflammatory cells in AOSD patients of different genetic backgrounds. Thus, further work with larger sample sizes, and a genetic study, is required to evaluate the effect of CXCL13 on progression to the chronic articular pattern in AOSD patients.

The limitations of this study include the lack of a normal skin and follow-up skin biopsy in AOSD for comparison with chemokine expression. We did not compare these markers with other febrile disorders for diagnostic value of these markers, and had a relatively small sample size for subgroup analysis and comparison with follow-up samples. Further studies involving larger sample size are required for evaluating usefulness of these markers in AOSD patients with control groups of other febrile disorders such as sepsis.

## Conclusions

We found significantly higher levels of CXCL10 and CXCL13 in the serum from patients with active AOSD. We also found that most follow-up AOSD patients exhibited reduced CXCL10 and CXCL13 levels after improvement in disease activity. These results indicate that CXCL10 and CXCL13 may play important roles in the pathogenesis of the acute inflammatory process in AOSD, although the levels did not significantly differ among the various AOSD disease courses. This exploration of chemokine levels in AOSD not only improves our understanding of the role played by such materials in immunopathogenesis but also lays the foundation for development of novel therapeutic strategies, such as anti-CXCL10 antibody administration, for AOSD.

## Additional files

Additional file 1:
**The inflammatory cell percentages staining for various chemokines upon cutaneous histopathological examination.** All values are means ± standard deviations. *CXCL10* C-X-C motif chemokine 10, *CXCL13* C-X-C motif chemokine 13, *CXCR3* C-X-C chemokine receptor type 3. (PPTX 63 kb) Additional file 2:
**Correlations between inflammatory cell grading (CD4/CD8/CD68 staining) and the percentages of inflammatory cells staining for C-X-C motif chemokine 10 (CXCL10)/C-X-C chemokine receptor type 3 (CXCR3) /CXCL13.** Spearman’s correlations were calculated. (PPTX 45 kb)

## Abbreviations

ACR: American College of Rheumatology
ANA: anti-nuclear antibody
AOSD: adult-onset Still’s disease
AST: aspartate transaminase
CC-CCL2: chemokine ligand 2
CRP: C-reactive protein
CXCL8: C-X-C motif chemokine 8
CXCR3: C-X-C chemokine receptor type 3
ELISA: enzyme-linked immunosorbent assay
ESR: erythrocyte sedimentation rate
HC: healthy controls
IFN-γ: interferon-γ
IL-1: interleukin-1
RA: rheumatoid arthritis
RF: rheumatoid factor
RHS: reactive hemophagocytic syndrome
SLE: systemic lupus erythematosus
TNF-α: tumor necrosis factor-α
