# Supplementary material for: Association of CXCL10 and CXCL13 levels with disease activity and cutaneous manifestation in active adult-onset Still’s disease
Source: Arthritis Res Ther. 2015 Sep 19;17(1):260. doi: 10.1186/s13075-015-0773-4 (PMC4575437; doi:10.1186/s13075-015-0773-4)
Supplement: Additional file 1: — The inflammatory cell percentages staining for various chemokines upon cutaneous histopathological examination. All values are means ± standard deviations. CXCL10 C-X-C motif chemokine 10, CXCL13 C-X-C motif chemokine 13, CXCR3 C-X-C chemokine receptor type 3. (PPTX 63 kb) [file 13075_2015_773_MOESM1_ESM.pptx]

## Slide 1
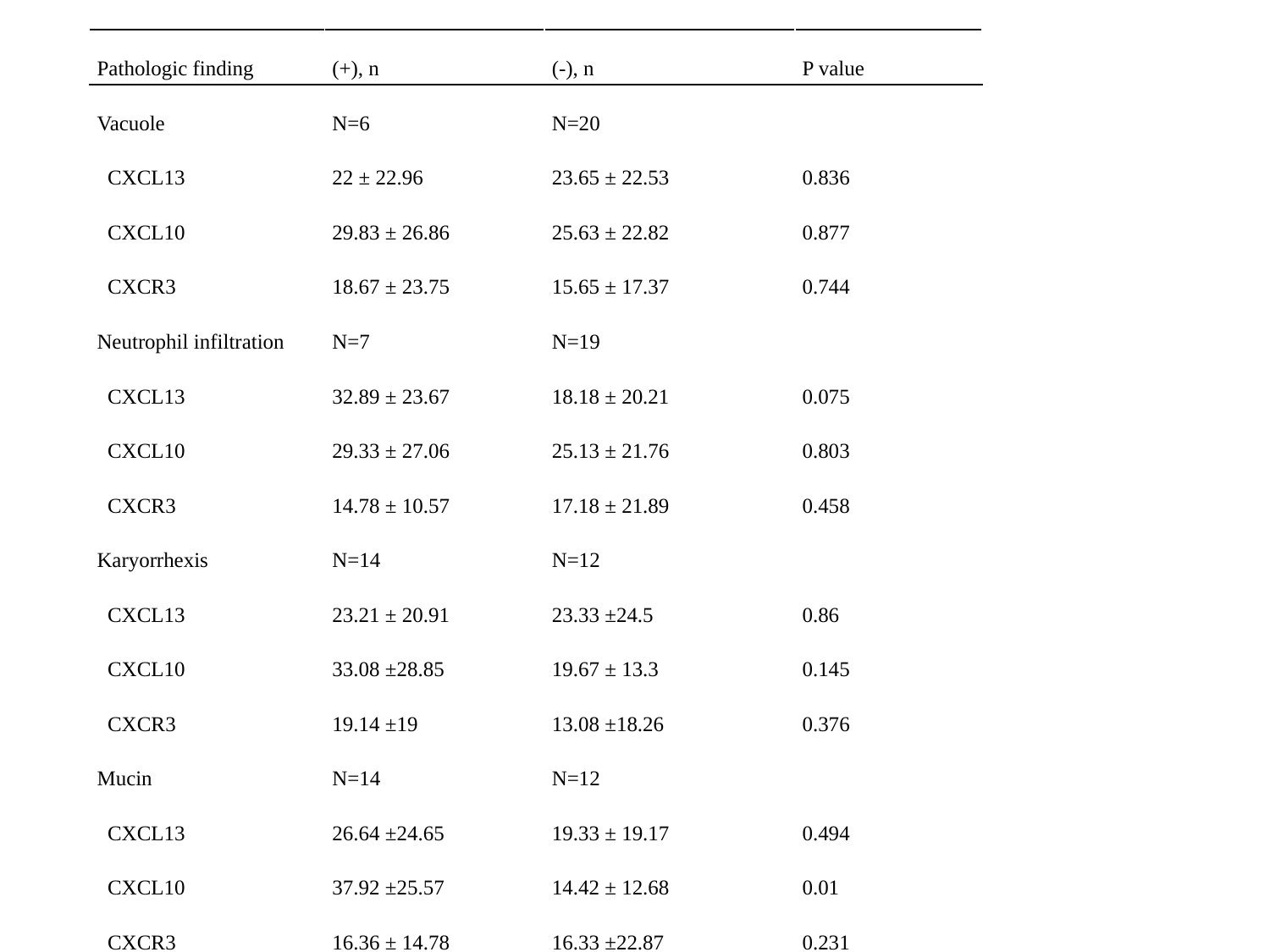

| Pathologic finding | (+), n | (-), n | P value |
| --- | --- | --- | --- |
| Vacuole | N=6 | N=20 | |
| CXCL13 | 22 ± 22.96 | 23.65 ± 22.53 | 0.836 |
| CXCL10 | 29.83 ± 26.86 | 25.63 ± 22.82 | 0.877 |
| CXCR3 | 18.67 ± 23.75 | 15.65 ± 17.37 | 0.744 |
| Neutrophil infiltration | N=7 | N=19 | |
| CXCL13 | 32.89 ± 23.67 | 18.18 ± 20.21 | 0.075 |
| CXCL10 | 29.33 ± 27.06 | 25.13 ± 21.76 | 0.803 |
| CXCR3 | 14.78 ± 10.57 | 17.18 ± 21.89 | 0.458 |
| Karyorrhexis | N=14 | N=12 | |
| CXCL13 | 23.21 ± 20.91 | 23.33 ±24.5 | 0.86 |
| CXCL10 | 33.08 ±28.85 | 19.67 ± 13.3 | 0.145 |
| CXCR3 | 19.14 ±19 | 13.08 ±18.26 | 0.376 |
| Mucin | N=14 | N=12 | |
| CXCL13 | 26.64 ±24.65 | 19.33 ± 19.17 | 0.494 |
| CXCL10 | 37.92 ±25.57 | 14.42 ± 12.68 | 0.01 |
| CXCR3 | 16.36 ± 14.78 | 16.33 ±22.87 | 0.231 |
