# Supplementary figures and images for: Association of CXCL10 and CXCL13 levels with disease activity and cutaneous manifestation in active adult-onset Still’s disease
Source: Arthritis Res Ther. 2015 Sep 19;17(1):260. doi: 10.1186/s13075-015-0773-4 (PMC4575437; doi:10.1186/s13075-015-0773-4)

## Slide 1
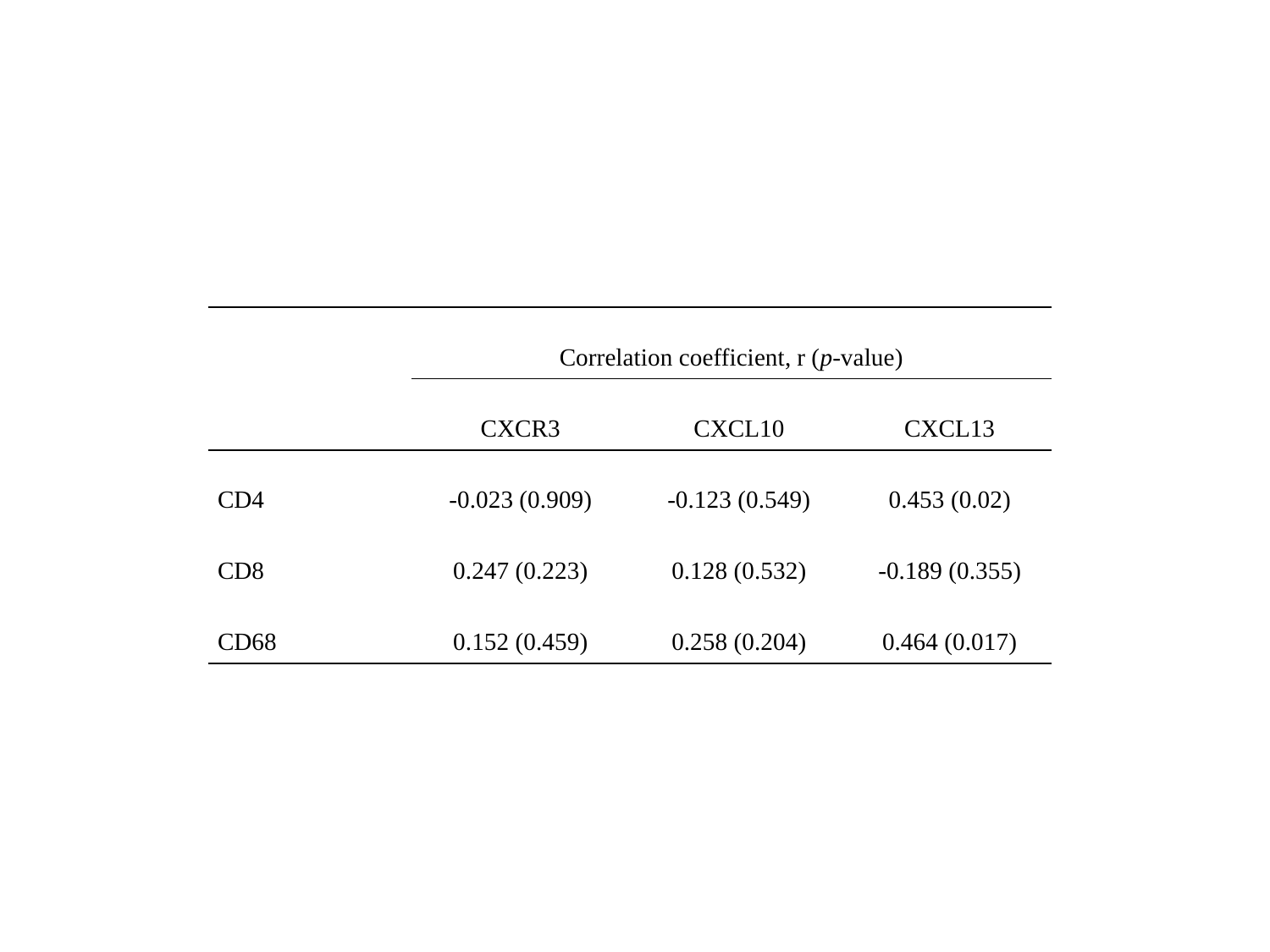

| | Correlation coefficient, r (p-value) | | |
| --- | --- | --- | --- |
| | CXCR3 | CXCL10 | CXCL13 |
| CD4 | -0.023 (0.909) | -0.123 (0.549) | 0.453 (0.02) |
| CD8 | 0.247 (0.223) | 0.128 (0.532) | -0.189 (0.355) |
| CD68 | 0.152 (0.459) | 0.258 (0.204) | 0.464 (0.017) |

Supplement: Additional file 2: — Correlations between inflammatory cell grading (CD4/CD8/CD68 staining) and the percentages of inflammatory cells staining for C-X-C motif chemokine 10 (CXCL10)/C-X-C chemokine receptor type 3 (CXCR3) /CXCL13. Spearman’s correlations were calculated. (PPTX 45 kb) [file 13075_2015_773_MOESM2_ESM.pptx]
